# Diagnostic and Prognostic Role of ^18^F-Fluoroestradiol PET in Metastatic Breast Cancer: The Second Youth of an Older Theranostic Concept

**DOI:** 10.3390/jcm11133589

**Published:** 2022-06-22

**Authors:** Francesco Fiz, Gianluca Bottoni, Giorgio Treglia, Pierpaolo Trimboli, Arnoldo Piccardo

**Affiliations:** 1 Nuclear Medicine Department, E.O. “Ospedali Galliera”, 16128 Genoa, Italy; gianluca.bottoni@galliera.it (G.B.); arnoldo.piccardo@galliera.it (A.P.); 2 Clinic of Nuclear Medicine, Imaging Institute of Southern Switzerland, Ente Ospedaliero Cantonale, 6500 Bellinzona, Switzerland; giorgio.treglia@eoc.ch; 3 Department of Nuclear Medicine and Molecular Imaging, Lausanne University Hospital, University of Lausanne, 1011 Lausanne, Switzerland; 4 Faculty of Biomedical Sciences, Università della Svizzera Italiana, 6900 Lugano, Switzerland; pierpaolo.trimboli@eoc.ch; 5 Clinic of Endocrinology and Diabetology, Lugano and Mendrisio Regional Hospital, Ente Ospedaliero Cantonale, 6500 Bellinzona, Switzerland

Since the discovery of the role of female hormones in breast cancer (BC) pathophysiology, in vivo detection of oestrogen receptor (ER) distribution has been one of the major goals of nuclear medicine and molecular imaging [1,2]. Hormone-blockade treatments represent, in fact, a safe and effective way to control ER-positive BC, even in the metastatic setting, and prevent recurrences [3,4,5]. Such an approach can be attempted in patients with evidence of ER on the clonal cell, which is estimated by biopsy or pathological examination of the primary tumour [6]. However, there is now ample evidence that ER expression might vary across disease sites, and there can be significant differences in this regard between primary tumour and distant metastases [7,8,9]. Since performing biopsies on remote disease localization might not always be feasible or even advisable, methods to obtain this information non-invasively have been sought after. The initial attempts in developing a molecular imaging tracer for scintigraphy imaging in the late 1970s were not successful, given the limited resolution of the method and the elevated background activity of some organs, such as liver [10,11]. However, some years later, the research group of Katzenellenbogen was able to develop a molecular imaging probe for PET devices, i.e., ^18^F-fluoroestradiol (FES), which was effectively the first radio-receptor positron-emitting tracer [12]. This tracer showed immediate promise; however, the related PET imaging method was then still in its infancy and, even later, the tracer landscape would continue to be dominated by fluorodeoxyglucose (FDG) for many years to come. It was only towards the end of the second decade of the 21st century that FES gained significant traction, leading to a marked increase of published research papers as well as its official approval, firstly in France (2016) and then in the United States of America (2020) [13].

The approval of FES-PET was grounded on its excellent capability of predicting the actual ER expression status on pathology; in a study by Chae and colleagues, among 37 patients with a positive FES-PET, all of them had oestrogen receptors on immunohistochemistry [14]. These data were recently confirmed by a larger, prospective trial, where FES-PET was able to predict the ER expression with very high sensitivity in the biopsied tumour lesions as well as in remote bone localizations [15]. In turn, the presence of ER is the only factor that can predict the effectiveness of endocrine therapy; tumour sites with poor or absent ER expression do not respond well to this approach [16,17]. Conversely, a widespread and intense FES positivity is linked with well-differentiated, ER-positive tumour forms, which have a significant chance to respond well to the endocrine treatment. In this sense, FES-PET represents a theranostic approach, directing the patients toward the most suitable therapy, while avoiding the costs and the potential side effects of an inadequate one [18].

It must be highlighted that, understandably, there are some caveats and limitations to this approach. First, the diagnostic potential of the method might vary, since physiological background activity might affect the detectability of tumour lesions; this issue is particularly marked in the liver, which presents the highest density of oestrogen receptors [19]. However, bone is the principal metastatic site of ER^+^ BC, which has a lower tendency to colonize visceral organs when compared with other BC subtypes [20]. Some factors, such as body mass index and level of sex hormone-binding globulin, may have a mild-to-moderate effect on FES uptake, whereas other factors, such as the menopausal status, do not [21]. Secondly, even if the method is very sensitive in determining the ER expression, this parameter represents a necessary, yet not per se sufficient, condition for the success of the endocrine treatment [16]. In fact, triggering of the ER-dependent intracellular mechanisms in BC can occur despite a pharmacological endocrine blockade, via functional alteration of intracellular domains or crosstalk with other pathways [22,23]. Consequently, even in the case of a positive FES-PET, the real effectiveness of a first-line endocrine treatment could vary across patients. Finally, especially in the metastatic setting, different cellular clones, with varying degrees of biological aggressiveness, might co-exist in the same patients. Particularly, an aggressive disease is signalled by the disappearance of ER on the cell and increased proliferation rate; in such a setting, the switch towards FDG-PET or a dual tracer (FES/FDG) PET is advised [24]. The FDG-positive disease is linked with a poorer prognosis and tends to not respond to endocrine treatment; in the case of mixed FDG- and FES-positive disease, the FDG/FES ratio, i.e., the measure of how prevalent the less differentiated component is, represents an important factor for predicting disease progression and patients’ overall survival [25].

Besides the identification of the aggressive clonal component, FDG-PET has an excellent sensitivity at the patient level, which is comparable to the one afforded by FES-PET [26]. However, FES-PET has better lesion-based sensitivity, especially in the restaging setting [26].

In conclusion, considering available evidence-based data [26,27,28], FES-PET proved to be a valid diagnostic, prognostic, and theranostic approach, which, after many years of preparation, is ready to take the main stage of differentiated cancer identification and treatment.
